# Metabolic pathway engineering for production of 1,2-propanediol and 1-propanol by *Corynebacterium glutamicum*

**DOI:** 10.1186/s13068-015-0269-0

**Published:** 2015-06-24

**Authors:** Daniel Siebert, Volker F. Wendisch

**Affiliations:** Chair of Genetics of Prokaryotes, Faculty of Biology and CeBiTec, Bielefeld University, Universitätsstr. 25, 33615 Bielefeld, Germany

**Keywords:** *Corynebacterium glutamicum*, Metabolic engineering, 1-propanol, 1,2-propanediol

## Abstract

**Background:**

Production of the versatile bulk chemical 1,2-propanediol and the potential biofuel 1-propanol is still dependent on petroleum, but some approaches to establish bio-based production from renewable feed stocks and to avoid toxic intermediates have been described. The biotechnological workhorse *Corynebacterium glutamicum* has also been shown to be able to overproduce 1,2-propanediol by metabolic engineering. Additionally, *C. glutamicum* has previously been engineered for production of the biofuels ethanol and isobutanol but not for 1-propanol.

**Results:**

In this study, the improved production of 1,2-propanediol by *C. glutamicum* is presented. The product yield of a *C. glutamicum* strain expressing the heterologous genes *gldA* and *mgsA* from *Escherichia coli* that encode methylglyoxal synthase gene and glycerol dehydrogenase, respectively, was improved by additional expression of alcohol dehydrogenase gene *yqhD* from *E. coli* leading to a yield of 0.131 mol/mol glucose. Deletion of the endogenous genes *hdpA* and *ldh* encoding dihydroxyacetone phosphate phosphatase and lactate dehydrogenase, respectively, prevented formation of glycerol and lactate as by-products and improved the yield to 0.343 mol/mol glucose. To construct a 1-propanol producer, the operon *ppdABC* from *Klebsiella oxytoca* encoding diol dehydratase was expressed in the improved 1,2-propanediol producing strain ending up with 12 mM 1-propanol and up to 60 mM unconverted 1,2-propanediol. Thus, B_12_-dependent diol dehydratase activity may be limiting 1-propanol production.

**Conclusions:**

Production of 1,2-propanediol by *C. glutamicum* was improved by metabolic engineering targeting endogenous enzymes. Furthermore, to the best of our knowledge, production of 1-propanol by recombinant *C. glutamicum* was demonstrated for the first time.

## Background

The usage of 1,2-propanediol ranges from building blocks in plastics industry, in de-icing and anti-freeze fluids, and as additive in cosmetics, nutrition, medicines, dyes, and liquid detergents [1]. Due to the very broad spectrum of applications of the bulk chemical 1,2-propanediol, also known as propylene glycol, annually over 1 billion pounds of 1,2-propanediol are sold in the United States and at least 1.2 million tons are consumed worldwide [2]. To date, most of this demand is accommodated by petrochemistry. In the main route, the steam cracking product propylene [3] is converted to propylene oxide [4, 5], which is further hydrolyzed to 1,2-propanediol [6]. The occurrence of toxic intermediates and side-products initiated efforts to find more sustainable and less toxic routes, e.g., by fermentation of renewable carbon sources by microorganisms. Various microorganisms showing potential to produce 1,2-propanediol from renewable feed stocks have been described, e.g., *Clostridium thermosaccharolyticum* [7], *Saccharomyces cerevisiae* [8, 9], *Escherichia coli* [1, 10], *Synechoccus elongates* [11], and *Corynebacterium glutamicum* [12].

The Gram-positive and *generally-recognized-as-safe* rod-shaped soil bacterium *Corynebacterium glutamicum* [13] is the main source of the worldwide production of the amino acids glutamate and lysine in a scale of over 5 million tons per year [14]. A wealth of information on *C. glutamicum* exists [14–18] including sequencing its genome [19] and creating a genome-streamlined chassis organism [20]. Metabolic engineering aimed at the production of not only many other amino acids [14, 21] but also for example at monomers of bioplastics (e.g., cadaverine [22, 23] and putrescine [23]), organic acids [24], carotenoids [25], and biofuels. *C. glutamicum* was engineered for isobutanol production and shown to exhibit less toxicity to isobutanol than *E. coli* [26, 27]. The isobutanol yield by recombinant *C. glutamicum* was competitive with *E. coli* [28]. In particular, overproduction of the biofuel ethanol under oxygen deprivation conditions is well-described for *C. glutamicum* and shown to be efficient [29–31]. Importantly, under these conditions *C. glutamicum* showed high tolerance to organic acid, furan, and phenolic inhibitors present in lignocellulose hydrolysates [30]. Thus, *C. glutamicum* is a promising alternative biofuel production host. To enable sustainable production from several alternative carbon sources, the substrate spectrum of *C. glutamicum* was widened by metabolic engineering [32]. Since 1,2-propanediol production by *C. glutamicum* has been shown [12] in principle, this study aimed at improving 1,2-propanediol production and at producing 1-propanol as derived compound. This primary alcohol, also named *n*-propanol, finds application in the solvent, cosmetic, and pharmaceutical industries, in antiseptic solutions, as precursor for diesel fuels and in the plastics industry and finally as biofuel [33–35]. *C. glutamicum* has previously been engineered for production of the biofuels ethanol [31] and isobutanol [26–28] but not for 1-propanol. Natural microorganisms are not known to secrete significant amounts of 1-propanol. However, *Propionibacterium freudenreichii* has been engineered for the direct conversion of propionyl-CoA to 1-propanol [34]. Engineered *E. coli* strains either convert 2-ketobutyrate to 1-propanol by variants of the threonine and citramalate pathways [36, 37] or by extending succinate dissimilation [35]. Finally, 1,2-propanediol can be converted in a two-step conversion to 1-propanol by diol dehydratase from *Klebsiella oxytoca* [33]. The latter pathway was chosen in this study for construction of a *C. glutamicum* 1-propanol-producing strain.

## Results

### Co-overexpression of *yqhD* from *E. coli* increased 1,2-propanediol production

*C. glutamicum* has previously been engineered for 1,2-propanediol production by expressing the heterologous genes *mgsA* and *gldA* encoding methylglyoxal synthase gene and glycerol dehydrogenase from *E. coli* [12]. Expression of these genes as artificial operon from the plasmid pEKEx3-*mgsA*-*gldA* in *C. glutamicum* WT yielded 19 ± 1 mM 1,2-propanediol within 51 h (Fig. 2) when using modified CGXII minimal medium with a decreased nitrogen content (5 g/L ammonium sulfate) and 184 ± 1 mM glucose as sole carbon source. Thus, the base strain produced 1,2-propanediol with a yield of 0.103 mol/mol glucose.

**Fig. 1:**
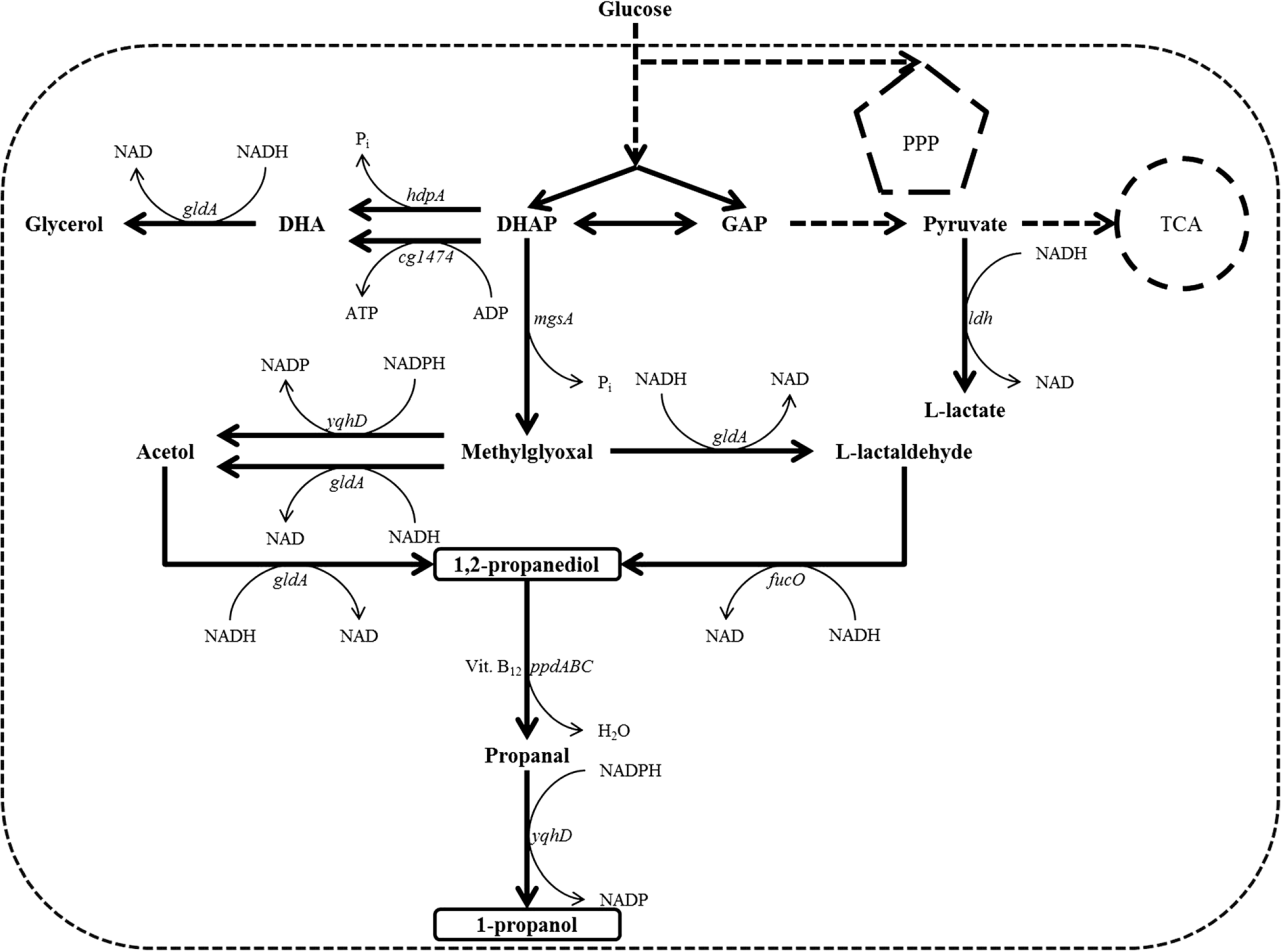
Scheme of the engineered metabolic pathway for the production of 1,2-propanediol and 1-propanol in *C. glutamicum*. Reactions are represented by *arrows* (preferred direction and cofactors), while *dashed lines* indicate multiple reaction steps. Genes coding for relevant enzymes are depicted next to the *arrows*: *cg1497*, predicted kinase related to dihydroxyacetone kinase; *hdpA*, dihydroxyacetone phosphate phosphatase (HdpA); *fucO*, propanediol oxidoreductase/lactaldehyde reductase (FucO); *gldA*, glycerol dehydrogenase (GldA); *ldh*, L-lactate dehydrogenase (LdhA); *mgsA*, methylglyoxal synthase (MgsA); *ppdABC*, diol dehydratase (PpdABC); *yqhD*, aldehyde reductase (YqhD). Abbreviations: *ADP* adenosine diphosphate, *ATP* adenosine triphosphate, *DHA* dihydroxyacetone, *DHAP* dihydroxyacetone phosphate, *GAP* glyceraldehyde 3-phosphate, *PPP* pentose phosphate pathway, *TCA* citric acid cycle, *Vit. B*_*12*_ vitamin B_12_

**Fig. 2:**
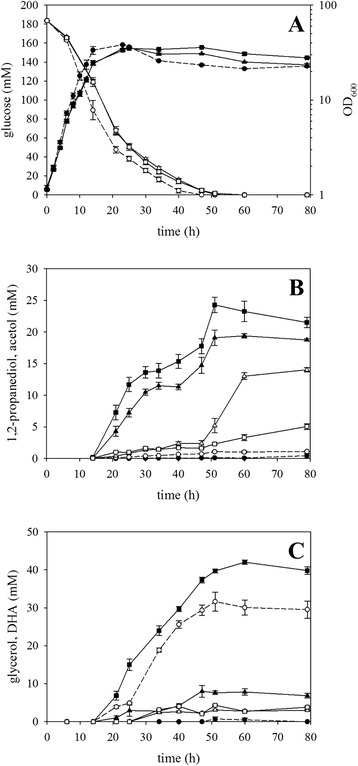
Influence of YqhD from *E. coli* on 1,2-propanediol production by recombinant *C. glutamicum* strains. Batch cultivation of *C. glutamicum* strains WT(pEKEx3) (*circles*, *dashed lines*), WT(pEKEx3-*mgsA*-*gldA*) (*triangles*, *solid lines*), and WT(pEKEx3-*mgsA*-*yqhD*-*gldA*) (*squares*, *solid lines*) were performed, and **a** optical density at 600 nm (*solid symbols*) and glucose concentration (*open symbols*), **b** 1,2-propanediol (*solid symbols*) and acetol (*open symbols*) concentrations, and **c** glycerol (*solid symbols*) and DHA (*open symbols*) concentrations are shown. Means and standard errors of three independent cultivations are shown

Methylglyoxal is a toxic intermediate of the conversion of dihydroxyacetone phosphate (DHAP) to 1,2-propanediol (Fig. 1), and in *E. coli*, additional overexpression of the alcohol dehydrogenase genes *yqhD* or *fucO* was shown to increase the yield of 1,2-propanediol from glycerol [10]. Heterologous expression of *yqhD* with *mgsA* and *gldA* from plasmid pEKEx3-*mgsA*-*yqhD*-*gldA* in *C. glutamicum* WT improved 1,2-propanediol production by about 27 % as 24 ± 1 mM 1,2-propanediol accumulated after 51 h (Fig. 2b), which correlated to a product yield of 0.131 mol/mol. Both *C. glutamicum* WT(pEKEx3-*mgsA*-*gldA*) and WT(pEKEx3-*mgsA*-*yqhD*-*gldA*) grew and utilized glucose as growth substrate slightly slower than the empty vector carrying control strain *C. glutamicum* WT(pEKEx3) (Fig. 2a). The addition of alcohol dehydrogenase gene *fucO* as fourth gene of the heterologously expressed operon on plasmid pEKEx3-*mgsA*-*yqhD*-*fucO*-*gldA* did not further improve 1,2-propanediol production as compared to WT(pEKEx3-*mgsA*-*yqhD*-*gldA*) (data not shown).

A comparison between strains WT(pEKEx3-*mgsA*-*gldA*) and WT(pEKEx3-*mgsA*-*yqhD*-*gldA*) with respect to by-product formation revealed that acetol, the direct precursor of 1,2-propanediol (Fig. 1), accumulated to higher concentrations in supernatants of WT(pEKEx3-*mgsA*-*gldA*) than of WT(pEKEx3-*mgsA*-*yqhD*-*gldA*), i.e., 14 mM as compared 5 mM, after glucose was depleted (Fig. 2b). On the other hand, WT(pEKEx3-*mgsA*-*gldA*) only produced 8 ± 1 mM glycerol as a by-product, whereas the additional expression of *yqhD* resulted in accumulation of 42 ± 1 mM (Fig. 2c). Interestingly, the empty vector control produced 32 ± 3 mM dihydroxyacetone (DHA), while *C. glutamicum* strains WT(pEKEx3-*mgsA*-*gldA*) and WT(pEKEx3-*mgsA*-*yqhD*-*gldA*) accumulated less than 5 mM DHA (Fig. 2c). Thus, preventing glycerol formation by the so far best producing strain WT(pEKEx3-*mgsA*-*yqhD*-*gldA*) offers the potential to improve 1,2-propanediol production.

### Stopping glycerol formation by deleting the gene *hdpA* resulted in higher yields of 1,2-propanediol

**Fig. 3:**
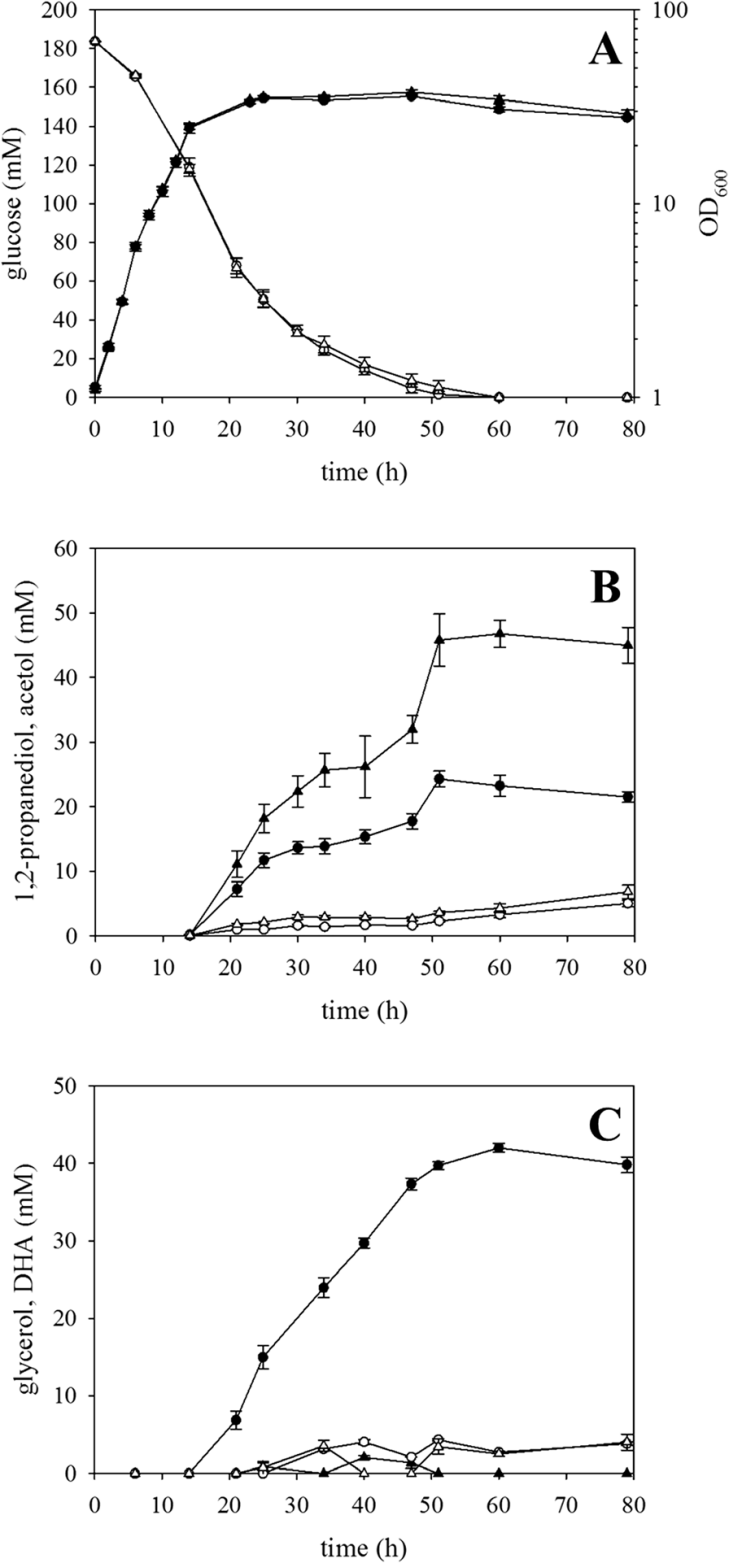
Influence of endogenous DHAP phosphatase HdpA on 1,2-propanediol production by recombinant *C. glutamicum* strains. Batch cultivation of *C. glutamicum* WT(pEKEx3-*mgsA*-*yqhD*-*gldA*) (*circles*) and Δ*hdpA*(pEKEx3-*mgsA*-*yqhD*-*gldA*) (*triangles*) were performed, and **a** optical density at 600 nm (*solid symbols*) and glucose concentration (*open symbols*), **b** 1,2-propanediol (*solid symbols*) and acetol (*open symbols*) concentrations, and **c** glycerol (*solid symbols*) and DHA (*open symbols*) concentrations are shown. Means and standard errors of three independent cultivations are shown

Typically, glycerol is hardly secreted by *C. glutamicum* WT, although two enzymes involved in glycerol formation have been found, namely *gpp*-encoded glycerol-3-phosphatase [38] and *butA*-encoded (S,S)-butanediol dehydrogenase [39]. In the experiments described above, glycerol was produced by the recombinant strains WT(pEKEx3-*mgsA*-*gldA*) and WT(pEKEx3-*mgsA*-*yqhD*-*gldA*) but nearly not by the parent strain WT(pEKEx3). This indicated that the heterologous enzymes present in these recombinants may be involved in glycerol formation. Since it is known that the *gldA*-encoded glycerol dehydratase from *E. coli* accepts also dihydroxyacetone, acetol, and methylglyoxal as substrates [40] (Fig. 1), it was tested if dihydroxyacetone formation can be prevented. Secretion of dihydroxyacetone by *C. glutamicum* WT occurs under certain conditions, e.g., acidic conditions [41], and was observed for WT(pEKEx3) under the conditions of 1,2-propanediol production described above. Two enzymes may be involved in DHA production, namely DHAP phosphatase encoded by *hdpA* [42] and a predicted kinase related to dihydroxyacetone kinases encoded by cg1497 [43]. To test if these enzymes are relevant for glycerol formation from DHA by the 1,2-propanediol-producing strain WT(pEKEx3-*mgsA*-*yqhD*-*gldA*), both genes were deleted by homologous recombination individually and in combination. The resulting strains *C. glutamicum* Δcg1497(pEKEx3-*mgsA*-*yqhD*-*gldA*), Δ*hdpA*(pEKEx3-*mgsA*-*yqhD*-*gldA*), and Δcg1497Δ*hdpA*(pEKEx3-*mgsA*-*yqhD*-*gldA*) were grown as described above for WT(pEKEx3-*mgsA*-*yqhD*-*gldA*). The deletion of the gene cg1497 had no impact on the 1,2-propanediol formation (data not shown). Upon deletion of *hdpA*, 1,2-propanediol production increased by about 90 % (Fig. 3b), while the double deletion mutant showed no further increase (data not shown). After 51 h of cultivation, *C. glutamicum* Δ*hdpA*(pEKEx3-*mgsA*-*yqhD*-*gldA*) accumulated 46 ± 4 mM 1,2-propanediol, which corresponds to a product yield of 0.249 mol/mol. *C. glutamicum* WT(pEKEx3-*mgsA*-*yqhD*-*gldA*) and Δ*hdpA*(pEKEx3-*mgsA*-*yqhD*-*gldA*) grew with comparable growth rates, utilized glucose comparably fast (Fig. 3a), and accumulated comparable concentrations (5 and 7 mM, respectively). However, glycerol was not a significant by-product (<5 mM) of the *hdpA* deletion strain, while the parental strain accumulated more than 40 mM glycerol (Fig. 3c). Thus, preventing DHA formation from DHAP by deletion of *hdpA* prevented subsequent formation of glycerol from DHA and improved 1,2-propanediol production.

### Deleting *ldh* prevented transient L-lactate accumulation and led to faster and higher 1,2-propanediol production

**Fig. 4:**
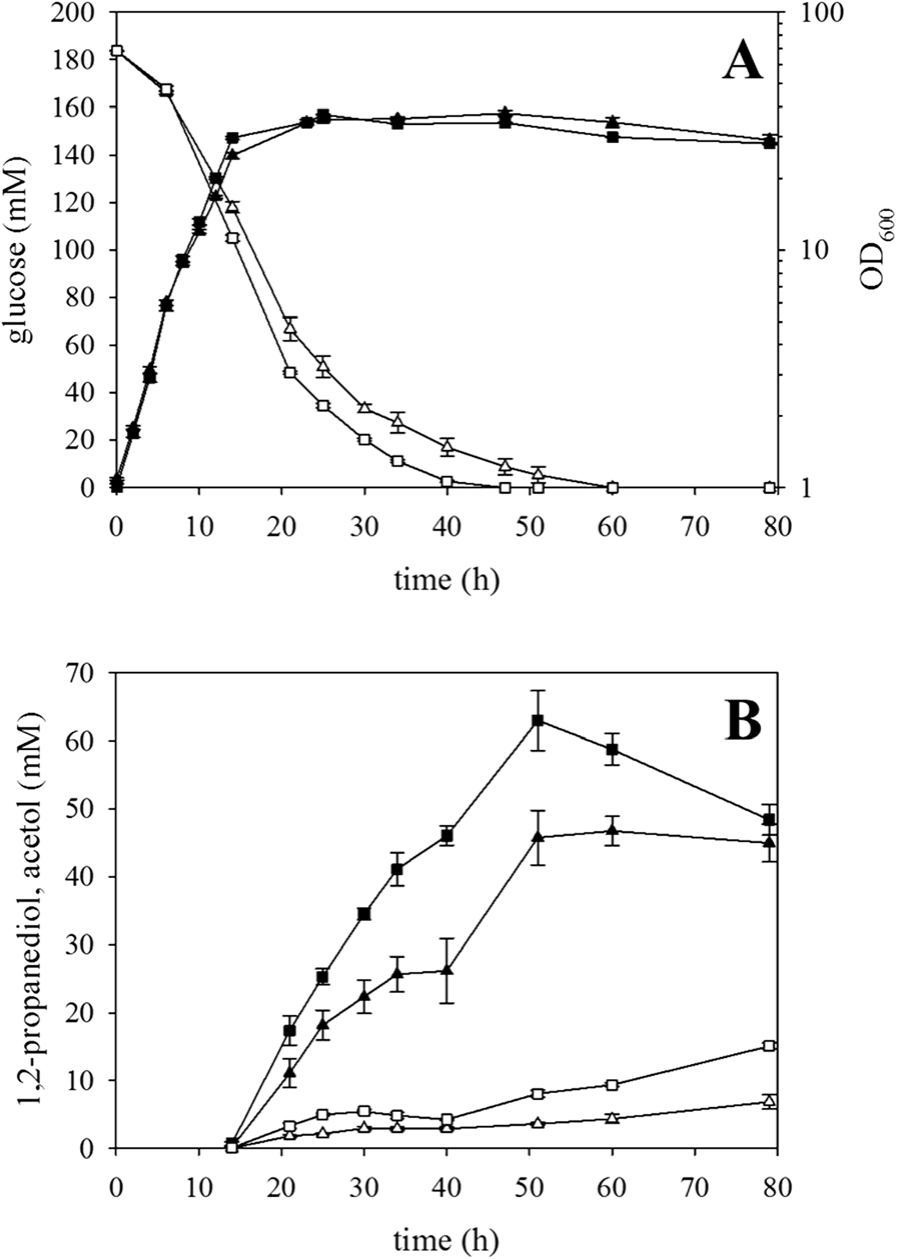
Influence of endogenous NADH-dependent L-lactate dehydrogenase Ldh on 1,2-propanediol production by recombinant *C. glutamicum* strains. Batch cultivations of *C. glutamicum* Δ*hdpA*(pEKEx3-*mgsA*-*yqhD*-*gldA*) (*triangles*) and Δ*hdpA*Δ*ldh*(pEKEx3-*mgsA*-*yqhD*-*gldA*) (*squares*) were performed, and **a** optical density at 600 nm (solid symbols) and glucose concentration (*open symbols*) and **b** 1,2-propanediol (*solid symbols*) and acetol (*open symbols*) concentrations are shown. Means and standard errors of three independent cultivations are shown

The deletion of *hdpA* prevented formation of about 40 mM glycerol but increased 1,2-propanediol accumulation by about 22 mM only (Fig. 3). Since 1,2-propanediol is more reduced than glycerol and since it is known that *C. glutamicum* utilizes excess NADH to reduce pyruvate to L-lactate, lactate formation may compete with 1,2-propanediol formation for NADH. In *C. glutamicum,* L-lactate is formed by fermentative, NADH-dependent lactate dehydrogenase LdhA under oxygen deprivation conditions [44] but transiently also during aerobic cultivation [45]. Re-uptake and re-utilization of lactate does not generate NADH but menaquinol, because both L- and D-lactate dehydrogenases LldD and Dld oxidize lactate to pyruvate in menaquinone-dependent reactions [45, 46]. Thus, *ldh* was deleted and the resulting strain *C. glutamicum* Δ*hdpA*Δ*ldh*(pEKEx3-*mgsA*-*yqhD*-*gldA*) was compared to strain Δ*hdpA*(pEKEx3-*mgsA*-*yqhD*-*gldA*) in batch cultivations. As consequence of introducing the *ldh* deletion, 1,2-propanediol production increased by about 38 %. *C. glutamicum* strain Δ*hdpA*Δ*ldh*(pEKEx3-*mgsA*-*yqhD*-*gldA*) accumulated 63 ± 4 mM 1,2-propanediol (Fig. 4b), which corresponds to a product yield of 0.343 mol/mol. Moreover, the *ldh* deletion strain utilized glucose faster and accumulated 1,2-propanediol faster than the parental strain, while the growth rates of both strains were comparable (Fig. 4a). Neither DHA nor glycerol accumulated to significant concentrations (<5 mM), but more acetol (15 mM as compared to 7 mM) was produced by the *ldh* deletion strain (Fig. 4b). Lactate formation by the *ldh* deletion strain was not detectable (<1 mM), while the parental strains and all other strains mentioned in Figs. 2, 3, and 4 accumulated lactate to low concentrations (between 1 and 4 mM) over the whole fermentation process. Taken together, *ldh* deletion improved 1,2-propanediol production considerably.

### Production of 1-propanol by recombinant *C. glutamicum*

**Fig. 5:**
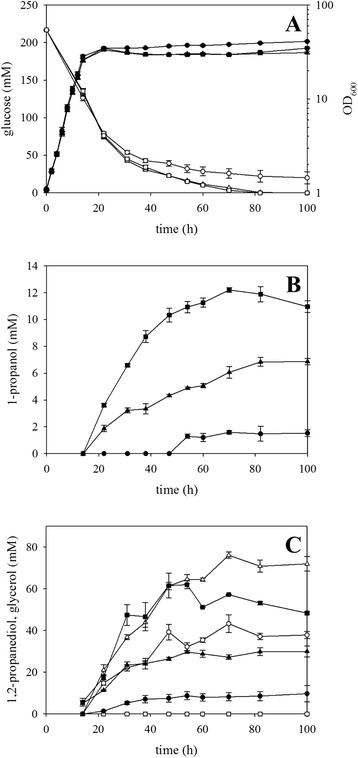
Production of 1-propanol by recombinant *C. glutamicum* strains. Batch cultivation of *C. glutamicum* WT(pEKEx3-*mgsA*-*gldA*)(pVWEx1-*ppdABC*) (*circles*), WT(pEKEx3-*mgsA*-*yqhD*-*gldA*)(pVWEx1-*ppdABC*) (*triangles*), and Δ*hdpA*Δ*ldh*(pEKEx3-*mgsA*-*yqhD*-*gldA*)(pVWEx1-*ppdABC*) (*squares*) were performed, and **a** optical density at 600 nm (*solid symbols*) and glucose concentration (*open symbols*), **b** 1-propanol concentrations, and **c** 1,2-propanediol (*solid symbols*) and glycerol (*open symbols*) concentrations are shown. Means and standard errors of three independent cultivations are shown

A 1,2-propanediol-producing *E. coli* strain produced 1-propanol when the *ppdABC* operon from *K. oxytoca*, which encodes a vitamin B_12_-dependent 1,2-propanediol dehydratase, was expressed [33, 47]. After vitamin B_12_-dependent 1,2-propanediol dehydratase has converted 1,2-propanediol to 1-propanal, the latter is reduced to 1-propanol by alcohol dehydrogenases such as YqhD [48]. Thus, the operon *ppdABC* of *K. oxytoca* was cloned into the expression vector pVWEx1, which is compatible with expression vector pEKEx3*,* and used to transform 1,2-propanediol-producing strains. Cultivated in minimal medium with 217 ± 1 mM glucose and 10 μM vitamin B_12_, *C. glutamicum* strain Δ*hdpA*Δ*ldh*(pEKEx3-*mgsA*-*yqhD*-*gldA*)(pVWEx1-*ppdABC*) accumulated 1-propanol to the highest concentration (12 ± 1 mM) after 70 h (Fig. 5a). This strain did not accumulate significant concentrations of glycerol, DHA, and acetol (data not shown). However, 1,2-propanediol was still the main product (62 ± 2 mM).

As expected from the 1,2-propanediol production experiments, deletions of genes *hdpA* and *ldh* were beneficial for 1-propanol production since strain WT(pEKEx3-*mgsA*-*yqhD*-*gldA*)(pVWEx1-*ppdABC*) accumulated almost twofold less 1-propanol (7 ± 1 mM) and 1,2-propanediol (30 ± 1 mM; Fig. 5b).

Strain WT(pEKEx3-*mgsA*-*gldA*)(pVWEx1-*ppdABC*) that did not overexpress *yqhD* from *E. coli,* which presumably is involved in reduction of 1-propanal to 1-propanol, only accumulated 2 ± 1 mM 1-propanol and utilized glucose incompletely (Fig. 5a). Accordingly, this strain only produced 9 ± 2 mM 1,2-propanediol and 43 ± 4 mM glycerol (Fig. 5c).

Taken together, 1-propanol was produced for the first time by recombinant *C. glutamicum* and strain Δ*hdpA*Δ*ldh*(pEKEx3-*mgsA*-*yqhD*-*gldA*)(pVWEx1-*ppdABC*) accumulated 1-propanol up to a concentration of 12 mM. Besides vitamin B_12_-dependent 1,2-propanediol dehydratase, also alcohol dehydrogenase YqhD appeared to be involved in converting 1,2-propanediol to 1-propanol.

## Discussion

In this study, production of 1,2-propanediol by *C. glutamicum* was improved and production of the biofuel molecule 1-propanol by *C. glutamicum* was shown for the first time. It has been shown previously that expression of the heterologous methylglyoxal synthase gene *mgsA* from *E. coli* was required for 1,2-propanediol and had to be coupled with glycerol dehydrogenase either encoded by heterologous gene *gldA* from *E. coli* or endogenous cgR_2242 [12]. Within 96 h, up to 25 mM 1,2-propanediol and 44 mM acetol were produced from 333 mM glucose as a sole carbon source [12]. Using a comparable strain but the cultivation setup employed in this study, it was possible to produce 19 mM 1,2-propanediol in 51 h from 184 mM glucose by overexpression of *mgsA* and *gldA* from *E. coli* in *C. glutamicum* WT (Fig. 2). Notably, accumulation of 1,2-propanediol and side products started after the cells entered the stationary phase, thus, production was not coupled to growth (Fig. 2).

Alcohol dehydrogenase YqhD proved beneficial for 1,2-propanediol production (increased by 27 % to a yield of 0.131 mol/mol glucose, Fig. 2), presumably because conversion of methylglyoxal to acetol and 1,2-propanediol was improved by YqhD. This enzyme has the following characteristics: a reductase activity for at least 12 aldehydes and thus increasing tolerance to aldehydes as aldehyde scavenger; preferring aldehydes over alcohols as substrates; a better conversion of alcohols longer than three carbon atoms; dependence of NADPH/NADP and divalent cations (e.g., zinc) as cofactors [48]. Notably, YqhD is NADPH-dependent [48] as compared to the NADH-dependent GldA, thus, YqhD is coupled to anabolic metabolism, which is driven by NADPH. Overexpression of *yqhD* proved beneficial for production of, e.g., 3-hydroxypropionic acid by *E. coli* [49], poly(3-hydroxypropionate) from glycerol using engineered *Klebsiella pneumoniae* [50], short-chain alcohols by *E. coli* [51], or acetol by *E. coli* [52].

Heterologous expression of *gldA* and *yqhD* from *E. coli* resulted in production of the side-product glycerol since these aldehyde reductases reduced DHA to glycerol [40]. Two possible enzymes were considered to be involved in the reduction of DHA metabolism, namely cg1497 and *hdpA* [42, 43]. Only the deletion of *hdpA* prevented glycerol formation and improved 1,2-propanediol production increasing the yield by about 90 % up to 0.249 mol/mol glucose (Fig. 3). The strain lacking endogenous *hdpA* showed improved 1,2-propanediol production due to two possible advantages. First of all, DHAP is not converted to DHA and, thus, supply of DHAP for the MgsA reaction to methylglyoxal was improved. Secondly, preventing reduction of DHA to glycerol increased provision of the redox cofactor NADH for the reactions converting methylglyoxal to 1,2-propanediol. Formation of glycerol as side-product of *C. glutamicum* strains expressing heterologous *gldA* and/or *yqhD* is distinct from glycerol production of *C. glutamicum* WT. In *C. glutamicum* WT, glycerol is formed from glycerol 3-phosphate by glycerol 3-phosphate phosphatase Gpp [38]. Since *C. glutamicum* WT secretes DHA under certain condition [41, 42], it is devoid of an enzyme catalyzing reduction of DHA to glycerol as efficient as observed in recombinants expressing heterologous *gldA* and/or *yqhD* from *E. coli*.

With the additional deletion of the gene *ldh*, it was possible to further increase the 1,2-propanediol production by about 38 % resulting in a yield of 0.343 mol/mol (Fig. 4). Deletion of *ldh* is a common strategy to improve production of organic acids under oxygen deprivation conditions [53, 54] since L-lactate is secreted by *C. glutamicum* under conditions of excess NADH. Two factors may have led to improved 1,2-propanediol production as result of *ldh* deletion. Firstly, provision of NADH for reduction of methylglyoxal to acetol and 1,2-propanediol is increased since pyruvate is not reduced to L-lactate. Secondly, pyruvate and possibly also other intermediates of glycolysis may accumulate as consequence of *ldh* deletion. This accumulation is plausible since deletion of pyruvate kinase Pyk led to accumulation of pyruvate and other glycolytic intermediates [55, 56]. In *E. coli*, methylglyoxal reacts spontaneously with glutathione to form a hemithioacetal, followed by detoxification by the glycoxalase system leading to the production of D-lactate [57]. *C. glutamicum* lacks glutathione but possesses mycothiol as its primary low molecular weight thiol [58]. A number of mycothiol-dependent reactions have been described for *C. glutamicum* including formaldehyde oxidation to formate [59, 60]. Although the reaction between mycothiol and methylglyoxal is currently not known in *C. glutamicum*, the overexpression of *mshA*-encoding mycothiol glycosyltransferase led to an increased robustness towards methylglyoxal [61].

Provision of NAD(P)H for reduction of acetol to 1,2-propanediol may still be limiting since even strain *C. glutamicum* Δ*hdpA*Δ*ldh* produced up to 15 mM acetol (Fig. 4). Notably, the accumulation of acetol increased after glucose was depleted while the 1,2-propanediol concentration decreased. Thus, 1,2-propanediol may be taken up again and oxidized to acetol to generate NADH, which may provide the cells with ATP in oxidative phosphorylation. Currently, it is not known whether oxidation of 1,2-propanediol occurs via the heterologous GldA from *E. coli* or by an endogenous enzyme. Interestingly, in a recombinant cyanobacterium producing 1,2-propanediol, alternative NADPH-alcohol dehydrogenases led to higher 1,2-propanediol titers, while acetol was not produced as side-product [11].

Additionally, the production of 1-propanol by *C. glutamicum* is reported for the first time in this study. Heterologous expression of the operon *ppdABC* from *K. oxytoca* encoding diol dehydratase in a 1,2-propanediol producing *C. glutamicum* strain was required for 1-propanol production of up to 12 mM (Fig. 5). Diol dehydratase PpdABC has the following characteristics: consisting of three subunits (α, β, and γ) with two units of a heterotrimer building the quaternary structure; indicated that the α- and γ-subunit promote the correct folding of each subunit; substrates are 1,2-propanediol, glycerol and 1,2-ethanediol with *Km* values of 0.08 μM, 0.73 mM, and 0.56 mM, respectively; lack of stereospecificity accepting (*R*)- and (*S*)-1,2-propanediol; dependent of adenosylcobalamin and divalent cations (e.g., potassium) as cofactors [62–64]. The observation that 1,2-propanediol was still the major product (up to 62 mM; Fig. 5) indicated that 1,2-propanediol to is not converted efficiently to 1-propanol by B_12_-dependent diol dehydratase PpdABC and YqhD. However, vitamin B_12_ may be limiting since it is not known if *C. glutamicum* can synthesize vitamin B_12_. In addition, provision of the cofactor NADPH may be a bottleneck.

There is potential for improving 1-propanol production with *C. glutamicum* as exemplified for *E. coli* [33, 47]. Overexpression of *ppdABC* in *E. coli* BW25113 for conversion of DHAP to 1,2-propanediol yielded 0.036 mol/mol 1-propanol from glucose [33], which is comparable to the yield of 0.032 mol/mol reported here (Fig. 5). The yield with *C. glutamicum* doubled as consequence of deleting *ldh* and *hdpA* (Fig. 5). Jain et al. (2014) optimized 1-propanol production by *E. coli* further [47]. The improvements included co-cultivation of one strain converting glucose to 1,2-propanediol and a second strain converting 1,2-propanediol to 1-propanol [47]. The first strain was improved by overexpressing an optimized gene set for conversion of DHAP to 1,2-propanediol and by deleting four genes to improve NADH provision [47]. Furthermore, heterologous expression of a gene coding for formate dehydrogenase and feeding the additional carbon source sodium formate and yeast extract improved the redox balance [47]. The second strain harbored a synthetic diol dehydratase gene cluster with optimized gene order (*ppdA-C-B*) and separation by linker sequences [47]. These metabolic engineering and medium optimization approaches may be helpful for improving 1-propanol production by the *C. glutamicum* strains described in this study. A number of engineering strategies to improve NADPH provision in *C. glutamicum* have been developed and include, e.g., transmembrane transhydrogenase PntAB [65], phosphoglucose isomerase mutants [66], NADPH-dependent glyceraldehyde-3-phosphate dehydrogenase [67], or NAD kinase [68]. Thus, production of 1-propanol may be increased further over the proof-of-concept established in this study.

## Conclusions

Metabolic engineering improved 1,2-propanediol production by *C. glutamicum*. Deletion of the endogenous genes *hdpA* and *ldh* combined with overexpression of the *E. coli* genes *mgsA*, *gldA*, and *yqhD* resulted in strain producing 1,2-propanediol from glucose in mineral salt medium with a product yield of 0.343 mol/mol. Further strain engineering led to strain capable of producing 1-propanol. This is the first report of 1-propanol production by recombinant *C. glutamicum*.

## Materials and methods

### Microorganisms, media, and cultivation conditions

In Table 1, all *C. glutamicum* strains and plasmids which were used for this study are presented. The *E. coli* strain DH5α [69] was used for the plasmid construction and was cultured in lysogeny broth complex medium (LB) [70]. Precultivation of *C. glutamicum* was performed in LB with 2 % glucose by inoculation from LB plates. For the main cultures of *C. glutamicum*, the cells of an overnight preculture were harvested by centrifugation (10 min; 3220 × g) and transferring the appropriate volume for an optical density (λ = 600 nm) (OD_600_) of 1 in 50-mL cultures. These cells were washed with CGXII minimal medium [71] without carbon source and without urea and ammonium sulfate. The cells were again centrifuged and resuspended with the same CGXII. As sole nitrogen source 5 g/L ammonium sulfate were added and as sole carbon source, glucose was used in the measured concentration given in the results. All cultivations of *C. glutamicum* were carried out in a volume of 50 mL in 500-mL baffled flasks at 30 °C and 120 rpm. The gene expression was induced by adding 1 mM isopropyl-β-D-thiogalactopyranoside (IPTG) at inoculation of the main culture. When appropriate, the medium was supplemented with 25 μg/mL kanamycin and 100 μg/mL spectinomycin. For 1-propanol production, it was necessary to add 10 μM of vitamin B_12_ to the medium. Growth was observed by measuring the OD_600_ using the V-1200 spectrophotometer (VWR International, Darmstadt, Germany) by diluting the samples into an OD_600_ range of 0.05–0.25. Additionally, 1-mL samples were taken at the time points given in the results and centrifuged (10 min; 16.000 × g), and the resulting supernatants were stored at −20 °C until further analysis.

**Table 1:** Strains and plasmids used in this study

Strain or plasmid	Relevant characteristics	Source or reference
*C. glutamicum* strains		
WT	Wild type (ATCC13032)	[76]
Δcg1497	In-frame deletion of cg1497 in *C. glutamicum* WT	This work
Δ*hdpA*	In-frame deletion of *hdpA* (cg2474) in *C. glutamicum* WT	This work
Δcg1497Δ*hdpA*	In-frame deletion of *hdpA* (cg2474) in *C. glutamicum* Δcg1497	This work
Δ*hdpA*Δ*ldh*	In-frame deletion of *ldh* (cg3219) in *C. glutamicum* Δ*hdpA*	This work
Plasmids		
pK19*mobsacB*	Kan^a^, mobilizable *E. coli* vector for the construction of insertion and deletion mutants of *C. glutamicum* (oriV, sacB, lacZ)	[75]
pEKEx3	Spec^a^; *C. glutamicum*/*E. coli* shuttle vector (P_tac_, *lacI* ^b^; pBL1, OriV_C.g._, OriV_E.c._)	[45]
pVWEx1	Kan^a^; *C. glutamicum*/*E. coli* shuttle vector for regulated gene expression (P_tac_, lacI^b^, pHM1519, oriV_C.g._, oriV_E.c._)	[77]
pK19*mobsacB*-Δcg1497	Kan^a^, pK19*mobsacB* with the deletion construct for cg1497	This work
pK19*mobsacB*-Δ*hdpA*	Kan^a^, pK19*mobsacB* with the deletion construct for *hdpA* (cg2474)	This work
pK19*mobsacB*-Δ*ldh*	Kan^a^, pK19*mobsacB* with the deletion construct for *ldh* (cg3219)	[28]
pEKEx3-*mgsA*-*gldA*	Derived from pEKEx3 for IPTG-inducible overexpression of *mgsA* and *gldA* from *E. coli* with artificial ribosome binding site in front of each gene	This work
pEKEx3-*mgsA*-*yqhD*-*gldA*	Derived from pEKEx3 for IPTG-inducible overexpression of *mgsA*, *yqhD*, and *gldA* from *E. coli* with artificial ribosome binding site in front of each gene	This work
pEKEx3-*mgsA*-*yqhD*-*fucO*-*gldA*	Derived from pEKEx3 for IPTG-inducible overexpression of *mgsA*, *yqhD*, *fucO*, and *gldA* from *E. coli* with artificial ribosome binding site in front of each gene	This work
pVWEx1-*ppdABC*	Derived from pEKEx3 for IPTG-inducible overexpression of *ppdABC* from *K. oxytoca* DSM4798 with artificial ribosome binding site in front of the gene cluster	This work

^a^Resistance gene

^b^Quantity

### Recombinant DNA work

All oligonucleotides used in this study were obtained from Eurofins MWG Operon (Ebersberg, Germany) or metabion international AG (Planegg, Germany) (Table 2). The plasmid construction was carried out with PCR fragments (KOD, Novagen, Darmstadt, Germany) generated with genomic DNA of *C. glutamicum* WT, *E. coli* DH5α (DNA preparation described by [72]), or *K. oxytoca* DSM4798 (DSMZ, Braunschweig, Germany) as template DNA. These fragments were cloned via Gibson Assembly [73] (enzymes provided by NEB, Frankfurt am Main, Germany) into the linearized vectors, and the resulting reaction was used for the transformation of *E. coli* DH5α cells using the calcium chloride method [70]. Therefore, pEKEx3 and pK19*mobsacB* were digested with the restriction enzyme *SmaI* and pVWEx1 with *BamHI* (Fermentas/Thermo Scientific, St. Leon-Rot, Germany). For the purification of the PCR fragments and the digested plasmids, the PCR purification kit or MinElute PCR purification kit (QIAGEN, Hilden, Germany) were applied. The plasmids were isolated from *E. coli* by using the QIAprep spin miniprep kit (QIAGEN, Hilden, Germany). All resulting vectors were sequenced to confirm the correctness of the cloned DNA fragments (SCF, CeBiTec, Bielefeld, Germany). The transformation of *C. glutamicum* was performed with electrocompetent cells [74] by electroporation [71] in a GenePulser Xcell™ plus PC Module (BioRad, München, Germany) but using LB with 2 % glucose in all stages of cultivation. All enzymes and kit systems were used like recommended in the manufacturer’s manuals.

**Table 2:** Oligonucleotides used in this study

Oligonucleotide name	Sequence (5′ **→** 3′)	Purpose
cg1497_upstrm_fw_pK19	*GACTCTAGAGGATCCCC*TTAACGCGCCGGGCTC	pK19*mobsacB*-Δcg1497
cg1497_upstrm_rv	*GGGTAGGTGATTTGAATTTGT*GCTTTCGGAACTGGACATAATCAGATAC	pK19*mobsacB*-Δcg1497
cg1497_dwnstrm_fw	*ACAAATTCAAATCACCTACCC*GGAATGGAGAATCTGGTAGAGATCGG	pK19*mobsacB*-Δcg1497
cg1497_dwnstrm_rv_pK19	*CGAGCTCGGTACCC*GAACTCTGGATGAGATAGCTGAGGTT	pK19*mobsacB*-Δcg1497
Dcg1497_fw_v3	CCACTGCCACGGAGCC	Verification of cg1497 deletion by PCR
Dcg1497_rv_v3	AACGAAGTGCCACTTCTTCCAC	Verification of cg1497 deletion by PCR
*nagD*_upstrm_fw_pK19	*GACTCTAGAGGATCCCC*TTCCCCGCAATGAGCCG	pK19*mobsacB*-Δ*hdpA*
*nagD*_upstrm_rv	*GGGTAGGTGATTTGAATTTGT*TGAAATGTTCACTGTCATAACACCATTGT	pK19*mobsacB*-Δ*hdpA*
*nagD*_dwnstrm_fw	*ACAAATTCAAATCACCTACCC*TTTCACGTACCAGATGAGCAGC	pK19*mobsacB*-Δ*hdpA*
*nagD*_dwnstrm_rv_pK19	*CGAGCTCGGTACCC*GGAACCTTCGGCTTGGATCTG	pK19*mobsacB*-Δ*hdpA*
D*nagD*_fw	GATGAACACGACCGTTGCC	Verification of *hdpA* deletion by PCR
D*nagD*_rv	GGGTGGTCTTTGAGGAGTTCTTC	Verification of *hdpA* deletion by PCR
*ldh*fow	TGATGGCACCAGTTGCGATGT	Verification of *ldh* deletion by PCR
*ldh*rev	CCATGATGCAGGATGGAGTA	Verification of *ldh* deletion by PCR
*mgsA*_fw_x3	*GACTCTAGAGGATCCCC* ***GAAAGGAGGCCCTTCAG***ATGGAACTGACGACTCGCACT	pEKEx3-*mgsA*-*gldA*, pEKEx3-*mgsA*-*yqhD*-*gldA*, pEKEx3-*mgsA*-*yqhD*-*fucO*-*gldA*
*mgsA*_rv_gld_DS	*TATCTCATAAAG*TTACTTCAGACGGTCCGCGA	pEKEx3-*mgsA*-*gldA*
*gldA*_fw_mgs_DS	*GGACCGTCTGAAGTAACTTTATGAGATA* ***GAAAGGAGGCCCTTCAG***ATGGACCGCATTATTCAATCACCG	pEKEx3-*mgsA*-*gldA*
*gldA*_rv_x3	*CGAGCTCGGTACCC*TTATTCCCACTCTTGCAGGAAAC	pEKEx3-*mgsA*-*gldA*, pEKEx3-*mgsA*-*yqhD*-*gldA*, pEKEx3-*mgsA*-*yqhD*-*fucO*-*gldA*
*mgsA*_rv	TTACTTCAGACGGTCCGCGA	pEKEx3-*mgsA*-*yqhD*-*gldA*
*yqhD*_fw_mgs	*GGACCGTCTGAAGTAA* ***GAAAGGAGGCCCTTCAG***ATGAACAACTTTAATCTGCACACCCC	pEKEx3-*mgsA*-*yqhD*-*gldA*
*yqhD*_rv	TTAGCGGGCGGCTTCGTATATA	pEKEx3-*mgsA*-*yqhD*-*gldA*
*gldA*_fw_yqh	*GCCGCCCGCTAA* ***GAAAGGAGGCCCTTCAG***ATGGACCGCATTATTCAATCACCG	pEKEx3-*mgsA*-*yqhD*-*gldA*
*mgsA*_rv_yqh_DS	*TATCTCATAAAG*TTACTTCAGACGGTCCGCGA	pEKEx3-*mgsA*-*yqhD*-*fucO*-*gldA*
*yqhD*_fw_mgs_DS	*GGACCGTCTGAAGTAACTTTATGAGATA* ***GAAAGGAGGCCCTTCAG***ATGAACAACTTTAATCTGCACACCCC	pEKEx3-*mgsA*-*yqhD*-*fucO*-*gldA*
*yqhD*_rv_gld_DS	*GAAATGAATAGC*TTAGCGGGCGGCTTCGTATATA	pEKEx3-*mgsA*-*yqhD*-*fucO*-*gldA*
*fucO*_fw_yqh_DS	*GCCGCCCGCTAAGCTATTCATTTC* ***GAAAGGAGGCCCTTCAGATG***ATGGCTAACAGAATGATTCTGAACG	pEKEx3-*mgsA*-*yqhD*-*fucO*-*gldA*
*fucO*_rv_gld_DS	*AAGGCAAGAATC*TTACCAGGCGGTATGGTAAAGCT	pEKEx3-*mgsA*-*yqhD*-*fucO*-*gldA*
*gldA*_fw_fuc_DS	*CATACCGCCTGGTAAGATTCTTGCCTT* ***GAAAGGAGGCCCTTCAG***ATGGACCGCATTATTCAATCACCG	pEKEx3-*mgsA*-*yqhD*-*fucO*-*gldA*
*ppdABC*_ko_fw_x1	*CTGCAGGTCGACTCTAGAG* ***GAAAGGAGGCCCTTCAG***ATGAGATCGAAAAGATTTGAAGCACTGG	pVWEx1-*ppdABC*
*ppdABC*_ko_rv_x1	*CGGTACCCGGGGATC*TTAATCGTCGCCTTTGAGTTTTTTACG	pVWEx1-*ppdABC*
*gldA*_Seq	GAACTGTGCTACAACACCCTG	Sequencing primer for *gldA*
*yqhD*_Seq	GTATTTGCCGTGCTCGATC	Sequencing primer for *yqhD*
*fucO*_Seq	GACCAATAAACCCAGTGTAC	Sequencing primer for *fucO*
*ppdABC*_Seq1	CGAACAGGAAACCACCGTTG	Sequencing primer for *ppdABC*
*ppdABC*_Seq2	ACGACCAGACCTTCACCCAC	Sequencing primer for *ppdABC*
*ppdABC*_Seq3	TACCTGCATACCTCCGCGAT	Sequencing primer for *ppdABC*
*ppdABC*_Seq4	AATCCTCCGACGTGGCCTTC	Sequencing primer for *ppdABC*
*ppdABC*_Seq5	CGAACAAGCACCCGGAATGG	Sequencing primer for *ppdABC*
pVWEx1_fw	CATCATAACGGTTCTGGC	Verification of correct pEKEx3/pVWEx1 derivatives by PCR/sequencing
pVWEx1_rv	ATCTTCTCTCATCCGCCA	Verification of correct pEKEx3/pVWEx1 derivatives by PCR/sequencing
M13_fw	CGCCAGGGTTTTCCCAGTCACGAC	Verification of correct pK19*mobsacB* derivatives by PCR/sequencing
M13_rv	AGCGGATAACAATTTCACACAGGA	Verification of correct pK19*mobsacB* derivatives by PCR/sequencing

Sequence in italics: overlapping sequences for Gibson-Assembly; sequence bold italics: artificial ribosome binding site

### Construction of *C. glutamicum* deletion strains

To delete the genes cg1497 and *hdpA* new plasmids were constructed by using the suicide vector pK19*mobsacB* [75]. For the deletion of cg1497, genomic regions flanking this gene were amplified via PCR from genomic DNA of *C. glutamicum* using the primer pairs cg1497_upstrm_fw_pK19/cg1497_upstrm_rv and cg1497_dwnstrm_fw/cg1497_dwnstrm_rv_pK19 (Table 2). The resulting PCR fragments were purified and cloned via Gibson-Assembly into the linearized vector pK19*mobsacB* resulting in the plasmid pK19*mobsacB*-Δcg1497 (Table 1). The deletion of the gene cg1497 was carried out with this plasmid by a two-step homologous recombination procedure described before [71]. For the verification of the correct in-frame deletion of the gene cg1497, a PCR (*Taq* DNA polymerase with ThermoPol® Buffer, NEB, Frankfurt am Main, Germany) was performed using the primer pair Dcg1497_fw_v3/Dcg1497_rv_v3 (Table 2). Accordingly the deletion of *hdpA* (cg2474) was realized, using the primer pairs *nagD*_upstrm_fw_pK19/*nagD*_upstrm_rv and *nagD*_dwnstrm_fw/*nagD*_dwnstrm_rv_pK19 (Table 2) for the cloning procedure of the plasmid pK19*mobsacB*-Δ*hdpA* (Table 1) and the primer pair D*nagD*_fw/D*nagD*_rv (Table 2) for the verification of the in-frame deletion via PCR. The plasmid pK19*mobsacB*-Δ*ldh* (Table 1) was already available [28]. Thus, the primer pair *ldh*fow/*ldh*rev (Table 2) was used to verify the successful in-frame deletion of *ldh* after the two-step homologous recombination.

### GC-MS measurements

The supernatants of the samples taken in the cultivation were analyzed using a TRACE GC ULTRA connected to an AS 3000 Auto-sampler and to an ISQ Single Quadrupole Mass Spectrometer using a TG-WAXMS (length: 30 m; I.D.: 0.25 mm; film: 0.25 μm) (Thermo Scientific, Dreieich, Germany). The thawed supernatants were directly diluted 1:10 with methanol (HPLC gradient grade; VWR Chemicals, Fontenay-sous-Bois, France) or after an additional 1:10 dilution step with water (*Milli-Q* grade). Prior to injection, the diluted samples were centrifuged (10 min; 16,000 × g) and the resulting supernatant was used for analysis. The operating setup was the following: the temperature of the MS transfer line and the ion source were hold at 230 °C; the injector temperature was set to 220 °C and a gradient was used for the oven (holding 40 °C for 1 min, increasing the temperature with a rate of 12 °C/min up to 230 °C and holding this for 5 min); in the constant flow mode, the flow rate of the carrier gas helium was 1 mL/min using the splitless mode of the injector (split flow: 10 mL/min; splitless time: 1.5 min; focus liner: 5 × 8 × 105 mm, splitless for 50-mm needle with glass wool); the electron impact ionization energy was 70 eV. The compounds 1,2-propanediol and acetol were measured with this method by creating a calibration curve with an external standard. The peaks were identified by retention time and were quantified using the intensity of one specific m/z value (1,2-propanediol: m/z = 45; acetol: m/z = 43). For the computational quantification, the program Xcalibur 2.1 (2.1.0 SP1.1160, Thermo Scientific, Dreieich, Germany) was employed.

### HPLC measurements

The compounds glucose, glycerol, DHA, lactate, propanal, and 1-propanol were quantified with a HPLC system (1200 series, Agilent Technologies, Böblingen, Germany). As a immobile phase, an organic acid resin column (300 × 8 mm) with the appropriate pre-column (40 × 8 mm) (Chromatographie-Service GmbH, Langerwehe, Germany) was installed and heated up to 60 °C while the mobile phase was 5 mM sulfuric acid in water (*Milli-Q* grade) with a flow rate of 0.8 mL/min or 1 mL/min. The signals were acquired with a refractive index detector (glucose, glycerol, propanal, and 1-propanol) and a diode array detector at a signal wavelength of 210 nm and a reference wavelength of 360 nm (DHA, lactate). For the calibration curve, external standards for every compound were prepared and the supernatants of the samples from the cultivations were measured undiluted after thawing.

## Abbreviations

Δ: deletion
ADP: adenosine diphosphate
ATP: adenosine triphosphate
butA: gene coding for (S,S)-butanediol dehydrogenase (ButA)
CeBiTec: Center for Biotechnology
cg1497: gene coding for predicted kinase related to dihydroxyacetone kinase
*C. glutamicum*: *Corynebacterium glutamicum*
CoA: Coenzyme A
cgR_2242: gene coding for putative aldo-keto reductase (AKR)
DHA(P): dihydroxyacetone (phosphate)
DNA: deoxyribonucleic acid
DSMZ: German Collection of Microorganisms and Cell Cultures
*E. coli*: *Escherichia coli*
*fucO*: gene coding for propanediol oxidoreductase/lactaldehyde reductase (FucO)
GC-MS: gas chromatography–mass spectrometry
gldA: gene coding for glycerol dehydrogenase (GldA)
gpp: gene coding for glycerol-3-phosphatase (Gpp)
hdpA: Gene coding for dihydroxyacetone phosphate phosphatase (HdpA)
HPLC: High-performance liquid chromatography
IPTG: isopropyl-β-D-thiogalactopyranoside
K*. oxytoca*: *Klebsiella oxytoca*
LB: lysogeny broth complex medium
ldh: gene coding for L-lactate dehydrogenase (LdhA)
mgsA: gene coding for methylglyoxal synthase (MgsA)
mshA: gene encoding mycothiol glycosyltransferase (MshA)
NADH and NAD: reduced or oxidized form of nicotinamide adenine dinucleotide, respectively
NADPH and NADP: reduced and oxidized form of nicotinamide adenine dinucleotide phosphate, respectively
NEB: New England Biolabs
OD_600_: optical density at wavelength (λ) 600 nm
PCR: polymerase chain reaction
PntAB: transmembrane transhydrogenase
*ppdABC*: operon coding for diol dehydratase (PpdABC)
PPP: pentose phosphate pathway
Pyk: pyruvate kinase
rpm: revolutions per minute
SCF: Sequencing Core Facility
TCA: citric acid cycle
Vit. B_12_: vitamin B_12_
WT: wild type
yqhD: gene coding for aldehyde reductase (YqhD)
